# Elucidating motion patterns in sperm cell motion with dynamic mode decomposition

**DOI:** 10.1007/s10867-026-09710-3

**Published:** 2026-03-27

**Authors:** Petr Šimánek, Jakub Hořenín, Islam S. M. Khalil, Veronika Magdanz, Anke Klingner, Alexander Kovalenko

**Affiliations:** 1 Faculty of Information Technology, Czech Technical University in Prague, Prague, 16000 Czech Republic; 2 RAM—Robotics and Mechatronics, University of Twente, Twente, 7522 NH The Netherlands; 3 Department of Systems Design Engineering, Waterloo Institute for Nanotechnology, University of Waterloo, Waterloo, N2L 3G1 ON Canada; 4 Department of Physics, German University in Cairo, Cairo, 11835 Egypt

**Keywords:** Dynamic mode decomposition, Periodical pattern analysis, Dynamical systems, Sperm motility, Biological microorganisms

## Abstract

The study employs dynamic mode decomposition (DMD) to elucidate the underlying mechanisms contributing to the enhanced motility observed in sperm bundles, primarily focusing on the role of flagellar synchronization. The decomposition reveals that synchronized flagellar movements might be a key factor enabling sperm cells to attain higher velocities when connected in bundles. Through DMD, periodical characteristics of individual periodical motion patterns, such as frequency, amplitude, and modal growth/decay rates (from DMD eigenvalues), are characterized, elucidating main parameters of the dynamic behavior of these biological systems, such as dominant frequencies of periodical motion, as well as amplitudes and velocities. The implications of this research extend beyond understanding sperm bundle dynamics, as the methodology is adaptable for identifying healthy sperm cells based on their motility patterns. Additionally, the approach holds potential for broader applications in studying other flagellar-driven microorganisms, providing a valuable tool for comparative analysis across various species.

**Supplementary Information:**

The online version contains supplementary material available at 10.1007/s10867-026-09710-3.

## Introduction

Collective effects in nature are a fascinating phenomenon observed across various scales and species, from microorganisms [1] to large animal groups [2]. This behavior is evident in the synchronized swimming of fish schools [3], the coordinated flight patterns of bird flocks [4], the marching of ant colonies [5], and the group movements of larger animals like wildebeests during migration [6].

Bacteria and other unicellular organisms use coordinated, flagella-driven movements through hydrodynamic interaction to swim collectively in the medium [7]. Such species also often exhibit collective motion in colonies [1], enhancing their ability to find nutrients and evade hostile environments [8]. The endeavor of a collective motion is dictated by the evolution and attributed to emergent properties of a moving body of individual units, that arise from the interactions and coordination between individuals, resulting in complex behaviors that benefit the group. These properties can include, for example, improved communication and information transfer [2, 9], enhanced environmental adaptation [2, 10], improved defense mechanisms [2, 11], enhanced foraging efficiency [12, 13], and more efficient motion in the medium [2, 4, 7, 14]. Analogous collective effects have also been reported for sperm cells in multiple species. In rodents (including mice), sperm cooperation and group swimming are relatively well established [15, 16].

In bovine sperm, several in vitro studies have reported the occurrence of connected pairs/small aggregates and examined their formation and motility [17–19]. In particular, Morcillo et al. reported that bull sperm bundles are predominantly two-cell bundles and that bundle occurrence increases with swimming duration (typically appearing 2–4 h after thawing), consistent with a collision-driven formation process modulated by head adhesion and hydrodynamic interactions [17]. Zhang et al. further quantified the transition from single to paired swimming and characterized changes in flagellar beating parameters during this transition [18]. While these observations support bundle formation as a plausible mechanism in bovine sperm under specific experimental conditions, we avoid interpreting it here as a universally established in vivo phenomenon.

To achieve a swimming velocity superior to that of a single cell, the flagellar motion in the bundle has to be synchronized. Synchronization of the flagella is broadly attributed to the problem of oscillator synchronization [20]; however, it is considerably more complex because both oscillations and beating patterns need to be coordinated. As there is no evidence of a chemical master oscillator that governs the molecular motors in multiple flagella, synchronizing appendages of the sperm bundles may be attributed to the hydrodynamic interactions between the flagella, similar to the multiflagellar organisms, such as bacteria [21–25] leading to an intricate dynamics of the system.

To explore dynamic components in motile bovine sperm cells, we apply DMD [26–29], which is a mathematical technique used for analyzing the dynamics of complex systems [30] through linear reduced-order models for high-dimensional complex data. It extracts spatial-temporal coherent structures from data, typically gathered from experiments or simulations [31]. DMD works by decomposing a sequence of data time stamps into modes, each associated with a specific frequency and growth/decay rate. These modes represent the underlying patterns and behaviors of the system, offering insights into its dynamics. This data-driven technique, rooted in fluid dynamics and systems theory, does not require *a priori* knowledge of the system’s governing equations, making it applicable to a wide range of systems. It effectively reduces high-dimensional data into fewer modes that capture the system’s essential dynamics. Additionally, in comparison with traditional machine learning techniques, DMD does not require a large amount of data, and the decomposition into modes with associated frequencies and growth rates provides clear insights into the dynamics and mechanisms driving the system. Finally, it can be applied to linear and nonlinear systems, stationary and non-stationary processes, and can handle noisy data to some extent.

DMD has found applications across various fields due to its versatility in handling nonlinear, high-dimensional data. In fluid dynamics, it helps in identifying vortex shedding and turbulent flows [32]. Environmental science uses DMD for weather pattern analysis and climate modeling [27]. It’s also applied in neuroscience to understand brain activity patterns, [31] finance for stock market trend analysis [33], and mechanical systems for vibration and control studies [34].

The knowledge gained from DMD analysis contributes to predictive models of sperm behavior and understanding of the underlying mechanisms that lead to sperm bundling. If sperm bundling is a beneficial sperm behavior that saves energy expenditure of single sperm and leads to more efficient sperm swimming, it will also give implications for improving sperm selection in assisted reproductive technologies and thus increase chances of successful fertilization. Moreover, elucidating motion patterns in the sperm cells allows a deeper understanding needed for improved design of multiflagellated sperm-inspired or sperm-templated microrobots [35, 36].

## Background

On the one hand, understanding the motion of sperm cells can benefit reproductive biology as it directly impacts the success of fertilization. Sperm cells are propelled by the beating of their flagella, which create the necessary thrust for movement through the viscoelastic fluid of the female reproductive tract. The motility patterns of sperm cells are influenced by various factors, including the presence of nutrients and activating agents, the viscosity of the surrounding fluid, and the presence of chemical gradients. Considering that sperm dedicate the majority of their energy expense towards motility [37], efficient motion is essential for sperm navigating complex environment, overcoming various physical barriers, and ultimately reaching the egg for fertilization. For this reason, motility is used as a common indicator of sperm quality and a predictor of fertilization success.

On the other hand, the importance of understanding sperm motility extends beyond basic biological interest. Insights into how sperm cells move can improve assisted reproductive technologies, such as *in vitro* fertilization and intracytoplasmic sperm injection, by enabling the selection of the highest quality sperm, according to factors important in natural sperm selection, in addition to being the most motile and viable sperm [38].

Single sperm cells exhibit various motility patterns, including linear, circular, and hyperactivated swimming [39]. In contrast, when opossum sperm cells form bundles, they exhibit a highly coordinated and efficient swimming pattern [40]. Sperm bundles are formed as a cooperative behavior that enhances the efficiency and effectiveness of sperm motility and migration. This natural strategy is influenced by factors such as incubation times and capacitation conditions [17]. Bundling behavior is particularly advantageous in competitive environments where high selection pressure exists, allowing for synchronized flagellar beating, which improves swimming efficiency compared to individual sperm cells.

As mentioned above, synchronized flagellar beating in sperm bundles is often attributed to hydrodynamic interactions [41] and physical contact between the cells, which can reduce energy expenditure and increase swimming efficiency. When sperm cells are in the immediate vicinity of each other, the movement of one cell’s flagellum affects the fluid around the neighboring cells, creating forces that may promote synchronization. Additionally, sperm cells may form head-to-head attachments due to adhesive regions. For bovine spermatozoa, the formation of mostly two-cell connected pairs and their transition dynamics have been reported, including changes in flagellar wave parameters during the transition [18].

The study of sperm motility and its underlying mechanisms has also inspired advancements in the field of microrobotics. Sperm-inspired and sperm-templated microrobots, such as IronSperm [35] and MagnetoSperm [36], mimic the propulsion mechanisms of sperm cells to navigate through fluid environments under weak rotating magnetic fields. These microrobots have potential applications in targeted drug delivery [42–44], minimally invasive surgery [45–48], and environmental monitoring [49, 50]. By understanding and replicating the efficient motility patterns of sperm bundles, researchers aim to develop more effective and versatile microrobots for a wide range of biomedical and industrial applications.

## Methodology

### The overall concept of dynamic mode decomposition

The mathematical concept of DMD can be described as follows:

Given a time-series sequence of data $\{{\textbf {x}}_1, {\textbf {x}}_2, \ldots , {\textbf {x}}_m\}$, where each datapoint ${\textbf {x}}_i \in \mathbb {R}^n$ represents the state of the system at time $t_i$, the goal of DMD is to approximate the dynamics governing the datapoints by a linear operator ${\textbf {A}}$, such that:

1 $$\begin{aligned} {\textbf {x}}_{i+1} \approx {\textbf {A}} x_i \end{aligned}$$

For the entire dataset, this can be represented in matrix form as:

2 $$\begin{aligned} {\textbf {X}}' \approx {\textbf {A}} {\textbf {X}} \end{aligned}$$

where ${\textbf {X}} = [{\textbf {x}}_1 \, {\textbf {x}}_2 \, \ldots \, {\textbf {x}}_{m-1}]$ and ${\textbf {X}}' = [{\textbf {x}}_2 \, {\textbf {x}}_3 \, \ldots \, {\textbf {x}}_m]$ are matrices constructed from the data snapshots.

The DMD algorithm involves the following steps:

1. Construct the data matrices ${\textbf {X}}$ and ${\textbf {X}}'$.
2. Compute the singular value decomposition (SVD) of ${\textbf {X}}$:
  3 $$\begin{aligned} {\textbf {X}} = {\textbf {U}} \boldsymbol{\Sigma } {\textbf {V}}^* \end{aligned}$$
  where *U* and *V* are unitary matrices, and $\Sigma$ is a diagonal matrix with ordered singular values.
3. Define the reduced-order representation of the operator **A** as $\tilde{{\textbf {A}}} = {\textbf {U}}^* {\textbf {A}}  {\textbf {U}}$, which can be approximated by:
  4 $$\begin{aligned} \tilde{{\textbf {A}}} = {\textbf {U}}^* {\textbf {X}}' {\textbf {V}} \boldsymbol{\Sigma }^{-1} \end{aligned}$$
  which can be derived from the relation $\textbf{X}' \approx \textbf{A}\,\textbf{X}$ and substitution the Moore–Penrose pseudo-inverse $\textbf{X}^+ = \textbf{V}\,\Sigma ^{-1}\,\textbf{U}^*$ to obtain the (4). Projecting this approximation onto the $r$-dimensional POD (proper orthogonal decomposition) subspace spanned by the leading $r$ left singular vectors $\textbf{U}$ via $\widetilde{\textbf{A}} = \textbf{U}^*\,\textbf{A}\,\textbf{U}$ then yields:
  5 $$\begin{aligned} \widetilde{\textbf{A}} = \textbf{U}^* \bigl (\textbf{X}'\,\textbf{V}\,\Sigma ^{-1}\,\textbf{U}^*\bigr )\,\textbf{U} = \textbf{U}^*\,\textbf{X}'\,\textbf{V}\,\Sigma ^{-1}, \end{aligned}$$
  which is the compact expression used for the reduced-order DMD operator.
4. Compute the eigenvalues and eigenvectors of $\tilde{A}$:
  6 $$\begin{aligned} \tilde{{\textbf {A}}} {\textbf {W}} = {\textbf {W}} \boldsymbol{\Lambda } \end{aligned}$$
  where *W* is the matrix of eigenvectors, and $\Lambda$ is a diagonal matrix containing the eigenvalues $\lambda _i$. Reconstruct the DMD modes in the original high-dimensional space:
  7 $$\begin{aligned} \boldsymbol{\Phi } = {\textbf {X}}' {\textbf {V}} \boldsymbol{\Sigma }^{-1} {\textbf {W}} \end{aligned}$$
  The columns of $\Phi$ are the DMD modes, and the eigenvalues $\lambda _i$ correspond to the growth rates and frequencies of the modes. Namely, the real part of $\log (\lambda _i)/\Delta t$ (where $\Delta t$ is the time step between snapshots) represents the growth or decay rate, while the imaginary part represents the oscillation frequency.

### Interpretation of individual DMD components

As mentioned above, DMD stands out from many machine learning techniques, with its interpretability as each component is associated with specific spatial structures and temporal behaviors:

#### DMD modes $(\phi _k)$

DMD modes are spatial structures that represent coherent features and the underlying spatial dynamics in the observed data. In classical fluid-mechanics applications, this data is a flow field; in our case, it is a time-resolved image field (the preprocessed, aligned sperm-region frames described in Sec. 3.1 and below). Each mode $\phi _k$ highlights dynamically significant regions in that image field (e.g., the flagellum region and head region) and their dominant spatiotemporal variations. By examining the spatial distribution of each mode, we can elucidate motion-related patterns that are otherwise problematic to detect:

8 $$\begin{aligned} \Phi = \{\phi _1, \phi _2, \ldots , \phi _r\} \end{aligned}$$

#### DMD eigenvalues $(\lambda _k)$

DMD eigenvalues are complex numbers that describe the temporal behavior of the corresponding DMD modes. Each eigenvalue $\lambda _k$ indicates how the associated mode evolves over time:

9 $$\begin{aligned} \boldsymbol{\Lambda } = \text {diag}(\{\lambda _1, \lambda _2, \ldots , \lambda _r\}) \end{aligned}$$

The real part of the eigenvalue ($\text {Re}(\lambda _k)$) indicates the growth or decay rate of the mode. Positive real parts indicate that the mode is growing over time, while negative real parts indicate that the mode is decaying. The imaginary part of the eigenvalue ($\text {Im}(\lambda _k)$) corresponds to the oscillation frequency, helping in identifying periodic behaviors or resonant frequencies in the flow.

($\text {Im}(\lambda _k)$) directly represents the DMD angular frequency $\omega$ of the oscillation. Therefore, the DMD frequency of the model can be calculated by [51]:

The relationship between discrete-time eigenvalues $\lambda _k$ and continuous-time eigenvalues $\mu _k$ is given by:

10 $$\begin{aligned} \mu _k = \frac{\log (\lambda _k)}{\Delta t} \end{aligned}$$

where $\log (\lambda _k)$ is the complex logarithm of the discrete-time eigenvalue and $\Delta t$ is the time interval between snapshots.

The real part of a continuous-time eigenvalue $\text {Re}(\mu _k)$ indicates the exponential growth ($\text {Re}(\mu _k) > 0$) or decay ($\text {Re}(\mu _k) < 0$) rate of the mode. The imaginary part $\text {Im}(\mu _k)$ represents the continuous oscillation frequency of the mode.

11 $$\begin{aligned} f = \frac{\text {Im}(\lambda _k)}{2\pi } \end{aligned}$$

#### Growth/damping rates $(\text {Re}(\lambda _k))$

Denoted as a real part of $\lambda$, growth/damping rates determine whether a mode is amplifying or attenuating over time. Growth rates help in identifying unstable features in the flow, which could lead to turbulence or other dynamic changes, while damping rates indicate which features are dissipating and becoming less significant over time.

#### Oscillation frequencies $(\text {Im}(\lambda _k))$

The imaginary part of $\lambda$ eigenvalue denotes oscillation frequencies, which are useful for detecting cyclical or periodic phenomena within the flow. This can be linked to physical phenomena such as vortex shedding, wave propagation, or other cyclic behaviors. In the present case, it identifies the periodical beating of flagella in sperm cells.

#### Reconstructed image field

Using the DMD modes and eigenvalues, we can reconstruct the observed image field at any given time point, which provides a visual and quantitative means to study the evolution of the sperm-region frames over time. It enables the prediction of future states of the image field based on current and past data, which is useful for forecasting and control applications.

12 $$\begin{aligned} \hat{\boldsymbol{X}}(t) = \boldsymbol{\Phi \Lambda }^t {\textbf {b}} \end{aligned}$$

where **b** is a vector of initial amplitudes.

#### Mode amplitudes

The mode amplitudes define the initial scaling factors for each DMD mode when reconstructing the flow field and give a measure of the relative importance or energy of each mode at the initial time. Amplitude modes are directly proportional to the impact on the overall flow dynamics:

13 $$\begin{aligned} {\textbf {b}} = \{b_1, b_2, \ldots , b_r\} \end{aligned}$$

where $b_r$ are individual amplitude components of the vector **b**.

### DMD for sperm cell videos

In this work, the analyzed *motility pattern* is the time-varying shape and intensity/contrast distribution of a sperm cell (or bundle) in a cropped, preprocessed image sequence. Since the region of interest contains the sperm head and flagellum, these patterns primarily reflect flagellar beating and head/whole-cell rotation as captured by phase-contrast imaging; the DMD input does not use tracked head positions or velocity time series directly.

For reproducibility, we explicitly describe how the DMD snapshots $\textbf{x}_i$ are constructed from the raw videos. Starting from the original microscopy video, we detect the target sperm cell or bundle in each frame and crop the corresponding bounding box (ROI). The ROI frames are then preprocessed as described in Sec. 4.2 (denoising/super-resolution as needed, rotation/alignment, and binarization/contrast enhancement). Finally, each preprocessed ROI frame is converted into a column vector by stacking its pixel values in a fixed order (row-major). Concretely, if the ROI image at time $t_i$ is an array $\textbf{I}_i\in \mathbb {R}^{H\times W}$ (after resizing/padding to a fixed $H\times W$ across all frames of a sequence), then the DMD snapshot is

14 $$\begin{aligned} \textbf{x}_i = \textrm{vec}(\textbf{I}_i)\in \mathbb {R}^{n},\qquad n=H\,W. \end{aligned}$$

Thus, DMD is applied to a time series of vectorized cropped bounding-box images (not the full microscope frame and not extracted contours only).

In this image-based setting, each DMD mode $\phi _k\in \mathbb {C}^{n}$ can be reshaped back to an $H\times W$ image and interpreted as a spatial pattern describing where the ROI tends to brighten/darken (or switch between foreground/background for binarized inputs) as that mode evolves in time. For sperm ROIs, dominant modes typically localize to the flagellum and head regions; therefore, oscillatory modes can be associated with recurrent image-space deformations driven by flagellar bending waves and/or head rotation, rather than a directly measured mechanical variable.

The mode amplitudes $b_k$ scale the contribution of each mode in reconstructing the image sequence. Because our snapshots are built from pixel values, $b_k$ has the same unit as the underlying pixel intensity. In practice, this is an arbitrary intensity unit (a.u.) determined by camera acquisition and preprocessing; for binarized images, it is dimensionless. Accordingly, amplitudes are interpreted comparatively within the dataset (relative contribution in reconstruction), rather than as an absolute physical unit.

For the experiments, we utilized PyDMD, a Python library specifically designed for performing Dynamic DMD and its various extensions. This library provides a flexible framework for decomposing the time-evolving data of complex systems into dynamic modes, which are spatial structures associated with specific temporal behaviors.

To find the optimal parameters for the DMD model, we conducted a grid search over a set of predefined hyperparameters. The DMD models tested included Sparsity-Promoting DMD (SpDMD) [52], Forward-Backward DMD (FbDMD) [53], Higher-Order DMD (HODMD) [54], and Extended DMD (EDMD) [28]. Each model offers unique features tailored to different aspects of data dynamics and noise handling, providing a basis for comparison.

The hyperparameters varied in the grid search included the singular value decomposition (SVD) ranks, which were tested across a range of values from 0 to 30. These ranks influence the dimensionality reduction step in the DMD process, impacting the balance between model complexity and noise reduction. Additionally, we explored total least square (TLS) ranks, with values $\{-1, 0, 1, 10, 20\}$. The TLS ranks aim to affect the robustness of the DMD models to noise by accounting for errors in both the input and output data.

Additionally, we examined the effects of the opt and exact parameters, testing them as either True or False. The opt parameter determines whether an optimization step is performed on the DMD modes to minimize residual error, potentially improving accuracy at the cost of increased computational complexity. The exact parameter specifies whether to use the exact DMD formulation, which avoids the SVD approximation and provides a more accurate representation of the system’s dynamics directly from the high-dimensional data matrix.

We also used the Intersection over Union (IoU) metric, calculated via the function, to further evaluate reconstruction accuracy.

Throughout the grid search, the best model configuration was identified and saved. The process involved iterating over all combinations of the specified hyperparameters for each DMD model class, fitting the models to the data, reconstructing the data, and calculating the reconstruction error using the IoU metric. The model configuration yielding the lowest error was recorded as the best.

Overall, the best results were obtained for SpDMD with the following parameters:
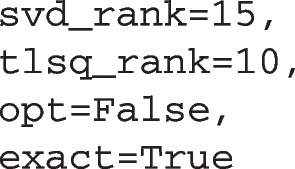


## Data

Cryopreserved bovine spermatozoa were obtained from Semex, Guelph, Canada. The samples were thawed for 2 min in a 37$^{\circ }$C water bath and then suspended in 2 mL of high-glucose Dulbecco’s Modified Eagle’s Medium (DMEM) supplemented with 4.5g/L glucose, sodium pyruvate, and sodium bicarbonate. The cell suspension was washed by centrifugation at 300 g for 5 min; the supernatant was removed, and the cells were resuspended in 2 mL fresh medium. To emulate a high-viscosity medium, 1% methylcellulose (Sigma Aldrich Nr. M0512) was added to the complete DMEM.

For high-speed videomicroscopy, 20 $\mu$L of the cell suspension was placed on microscope slides (0.18 mm $\times$ 60 mm $\times$ 24 mm) with coverslips attached using parafilm to form microchambers. The unstretched parafilm strips were placed between the microscope slide and cover slide and placed on a hot plate for a few seconds while being held down until the parafilm became transparent, indicating it has sealed the chamber. The chamber height was around 20 microns.

For imaging, 20 microliters of sperm suspension were loaded into the respective fluid (sperm medium with or without supplement of 1% methylcellulose). Bovine sperm cell density after thawing and washing was $10^7$ cells/mL, and was reduced to about $10^6$ cells/mL for imaging to avoid excessive density and crossing trajectories during recording. Videos were recorded immediately after washing, at room temperature and atmospheric pressure, using a Zeiss Axio Observer microscope equipped with phase contrast and a Basler high-speed camera. Recordings were made in phase-contrast mode with either a $20\times$ or $40\times$ objective lens. The frame rate was set at 100 frames per second (fps), with each video capturing at least 500 frames.

### Sperm cells/bundle detection

To avoid ambiguity in terminology, throughout this manuscript, we use the term *bundle* to denote two (or more) sperm cells that are physically attached (typically head-to-head) and therefore move as a single object over multiple consecutive frames. We use the term *group* (or *cluster*) to denote sperm cells that are in close proximity in the image plane but are not stably attached; such groups may separate or merge purely due to relative motion and overlapping projections.

For sperm cell detection, we used the proprietary tool, developed for individual sperm/bundle detection. Contour detection methods identify individual sperm cells, delineating their boundaries and associating them with bounding boxes. The kinematic summary of the analyzed trajectories (including the standard CASA velocities VCL, VSL, and VAP) is reported in Table 1.

**Table 1:** Speed results of selected sperm cells. Here, VCL denotes curvilinear velocity, VSL straight-line velocity, and VAP average-path velocity

Cell id	VCL ($\mu$m/s)	VSL ($\mu$m/s)	VAP ($\mu$m/s)
Single cell S-A	43.294	9.845	12.669
Single cell S-B	44.786	18.326	17.577
Single cell S-C	44.390	18.326	17.108
Single cell S-D	41.262	7.731	12.633
Single cell S-E	33.762	5.175	9.306
Single cell S-F	32.641	10.886	13.191
Single cell S-G	6.294	10.233	20.542
Bundle B-A	53.273	14.523	17.261
Bundle B-B	86.272	0.653	5.050
Bundle B-C	68.438	4.354	8.188
Bundle B-D	53.394	2.771	8.631
Bundle B-E	43.388	6.919	13.174
Bundle B-F	74.191	3.032	7.889

The tracking model integrates the Kalman filter to predict and refine cell trajectories over time. This method addresses challenges such as missed detections due to noise or overlapping objects. The Kalman filter, employing a constant velocity model, is used to maintain continuous tracking of sperm cells by estimating their positions even when some data is noisy or unavailable. Bounding box overlaps across successive frames are evaluated to link detected cells temporally, with an 80% overlap threshold indicating the persistence of the same cell. The system also accounts for scenarios like occlusion, collisions, and trajectory convergence, which are common in dense sperm samples. In scenarios where bounding boxes merge, the algorithm detects these transitions, recognizing instances of sperm cells forming bundles. The approach adapts to high-density environments, ensuring robust trajectory mapping despite potential overlaps or classification ambiguities. Representative examples of reconstructed cell trajectories are provided in the Supplementary Materials (Figs. S1–S3).

To distinguish between single sperm cells and bundles, a classification model based on ResNet18, pretrained on ImageNet, is utilized. The classifier categorizes bounding box contents into single sperm cells, bundles, groups of nearby cells, or other entities. Aggregating frame-by-frame classifications into trajectory-based insights helps identify transitions from individual cells to bundles. The rules for classification ensure accuracy, requiring at least 40% of frame-level classifications to label an object as a bundle. This methodology effectively isolates and characterizes single sperm cells and bundles, enabling precise quantification of their respective motility and behavioral patterns.

In total, we collected 13 video sequences: 7 videos of single sperm cells (further denoted as S-X) and 6 videos of bundles ((further denoted as B-X) Fig. 2) consisting of two or three sperm cells (bundle B in Fig. 2). All sperm cells displayed normal swimming behavior, as a non-capacitating medium was used to avoid inducing capacitation. The dataset encompasses a diverse set of motility patterns, capturing both individual and collective sperm cell dynamics.

### Data preprocessing

To enhance the quality of the recorded videos and prepare the data for analysis, several preprocessing steps were performed. Initially, the videos underwent denoising and super-resolution processing using pre-trained models available as open source. Specifically, we employed Real-ESRGAN [55], a super-resolution model known for its capability to upscale images while preserving realistic textures. For denoising, we used the pre-trained current state-of-the-art CascadedGaze model [56], which effectively removes noise from images without compromising structural details.

Following the enhancement of image quality, automatic sperm cell detection was conducted using Grounding DINO [57], an advanced object detection model based on the Swin Transformer architecture. The model was prompted with the term “sperm cell” and configured with a confidence threshold of 0.1 and an Intersection over Union (IoU) threshold of 0.5 to generate initial bounding box annotations. These annotations were then manually reviewed and adjusted using the Computer Vision Annotation Tool (CVAT) to ensure accuracy.

Subsequent preprocessing steps included cropping the bounding boxes around the detected sperm cells to focus on regions of interest, with a cell centered and crop size $150\times 150$ pixels ($\approx$ 68 µm). This centering and rotation mean the DMD captures body-frame flagellar dynamics rather than lab-frame translation or orientation changes. Direction detection and rotation were applied to align the sperm cells in a consistent orientation across frames, facilitating a more precise analysis of their motility patterns. Video binarization was performed to enhance the contrast between the sperm cells and the background, aiding in the extraction of morphological features.

**Fig. 1:**
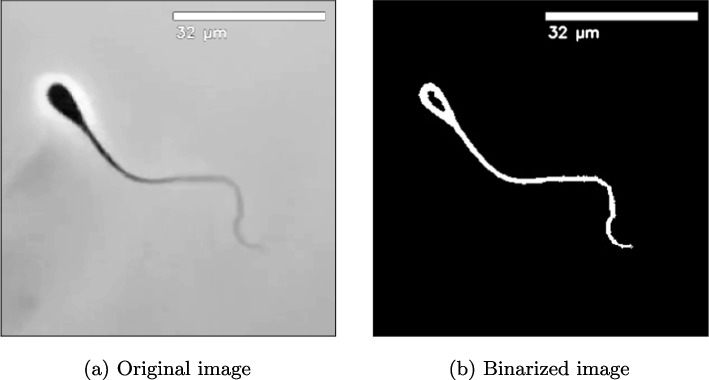
Comparison between original (left) and preprocessed (right) images of a sperm cell

Figure 1 illustrates the comparison between an original image frame and its corresponding preprocessed version after applying the aforementioned techniques.

**Fig. 2:**
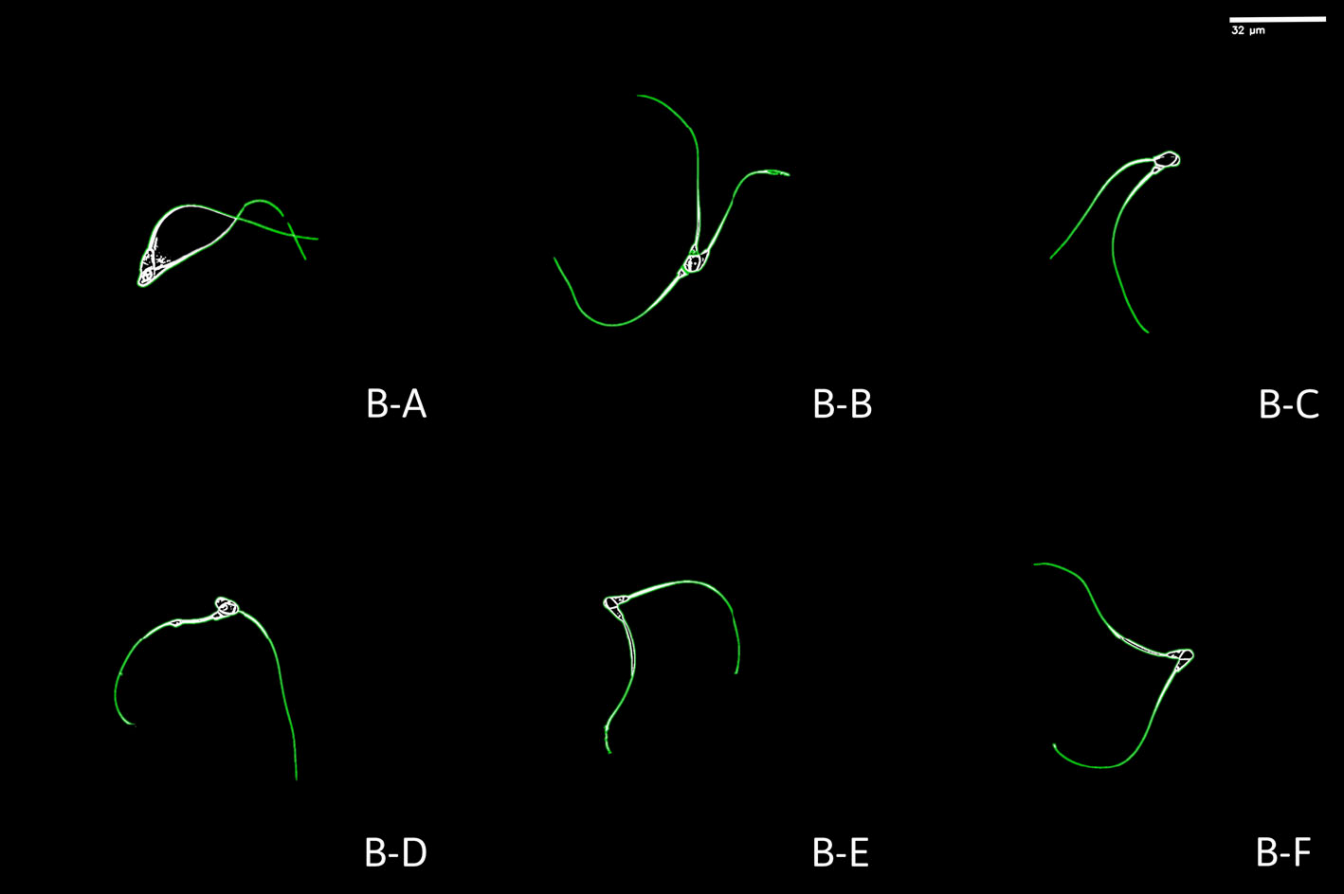
Binarized images of sperm cell bundles used in the experiment. Bundle B-B contains 3 sperm cells: two aligned in one direction, while the third one is aligned in the opposite direction. Other bundles contain two sperm cells

Two distinct behaviors emerged in our observations: (1) the occasional aggregation of sperm cells into bundles and (2) dynamic changes in image contrast causing a halo-like appearance around the sperm heads due to flagellar-driven rotation and the phase-contrast imaging modality. Bundle formation of sperm has been reported across multiple species (e.g., rodents and insects) [16, 58]. For bovine sperm in particular, bundle formation has been observed and quantified in vitro under both fresh and cryopreserved conditions [17, 18].

Because the experiments in the present study were performed on cryopreserved bovine spermatozoa, we cannot exclude that the observed attachments/aggregates are influenced by the in vitro preparation (e.g., freezing/thawing, medium composition, viscosity, and confinement in the microchamber). We therefore interpret the bundles shown in Fig. 2 as experimentally observed configurations consistent with prior in vitro reports, rather than as definitive evidence of an in vivo collective strategy in bovine sperm.

Bundle formation may arise from a combination of hydrodynamic and adhesive interactions, becoming especially pronounced after extended incubation periods. The alternation in head brightness results from the cells’ rotational motion changing the phase shift of transmitted light through the structure and thereby causing “in-phase” areas to appear brighter and “out of phase” areas to appear darker under phase-contrast microscopy.

The final dataset, comprising both enhanced image quality and precise annotations, was formatted for compatibility with further analysis using DMD. The preprocessing steps ensured that the data was of sufficient quality and consistency for accurate extraction of motility patterns.

### Ethical considerations

All experimental procedures were conducted under relevant guidelines and regulations for the use of animal-derived biological materials with approval from the University of Waterloo Research Ethics Board (44999). Bovine semen was kindly donated by Semex. No live animals were used directly in this study.

### Data availability

The processed data set, including the preprocessed videos and annotation files, is available upon reasonable request. This allows for the reproducibility of the results and facilitates further research in the analysis of sperm motility patterns.

## Results and discussion

### Analysis of motility patterns

The kinematic parameters (curvilinear velocity (VCL) and straight-line velocity (VSL) [59]) and DMD results suggest differences in motility patterns between the analyzed single sperm cells and sperm bundles. In this dataset, single cells exhibited moderate curvilinear velocity (VCL: 32.64–44.79 µm/s) and higher progressive motility, as reflected by their straight-line velocity (VSL: 5.18–18.33 µm/s). In contrast, the analyzed bundles tended to show higher VCL (43.39–86.27 µm/s) but lower VSL (0.65–14.52 µm/s), consistent with vigorous but less directional movement. For instance, bundle B had the highest VCL (86.27 µm/s) but the lowest VSL (0.65 µm/s), suggesting rapid yet irregular, strongly curvilinear (i.e., non-progressive) motion. Here, we use *irregular* in the descriptive sense (low directional persistence) and do not claim deterministic chaos in the nonlinear-dynamics sense (sensitivity to initial conditions). Notably, in our dataset bundle, A was an outlier, with VSL (14.52 µm/s) comparable to single cells.

#### Limitation (sample size and inferential statistics)

**Fig. 3:**
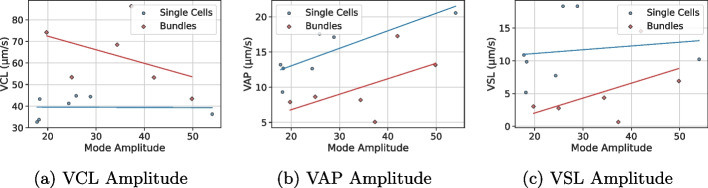
Scatter plots of mode amplitude versus (left) VCL, (middle) VAP, and (right) VSL for single cells (blue) and bundles (red). Lines indicate group-specific linear regressions

Importantly, the present study is based on 7 single-cell videos and 6 bundle videos (13 sequences in total). This limited sample size restricts statistical power and generalizability; therefore, all reported group differences should be interpreted as descriptive and preliminary, and we refrain from presenting formal hypothesis tests (e.g., t-tests/Mann–Whitney U tests) or correlation p-values. The primary contribution of this work is thus a proof-of-concept demonstration that DMD can extract interpretable oscillatory components from sperm-region image sequences and can highlight potentially meaningful differences between single cells and bundles, motivating follow-up studies on larger cohorts where inferential statistics will be appropriate (Fig. 3).

**Fig. 4:**
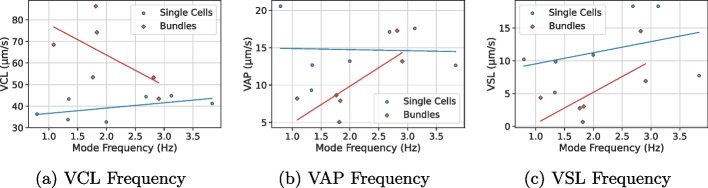
Scatter plots of mode frequency versus (left) VCL, (middle) VAP, and (right) VSL for single cells (blue) and bundles (red). Lines indicate group-specific linear regressions

#### DMD modes and frequency dynamics

**Fig. 5:**
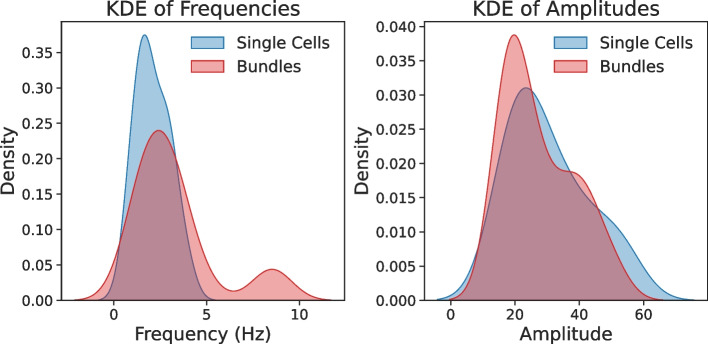
Kernel density estimation (KDE) of beat frequency (left) and mode amplitude (right) for single sperm cells (blue) and bundles (red), based on our DMD-derived frequencies and amplitudes

DMD analysis suggests differences in the oscillatory dynamics of the analyzed single cells and bundles. In this dataset, single cells exhibited dominant low-frequency modes (1.33–5.12 Hz) with moderate amplitudes (10.73–54.01), consistent with rhythmic flagellar beats that can support progressive motility. For example, single cell S-G showed a dominant low-frequency mode (0.795 Hz, amplitude=54.01) together with VSL = 10.23 µm/s. Bundles, however, displayed broader frequency ranges, including higher-frequency modes (e.g., 8.53 Hz in B-A), often paired with lower amplitudes (16.04–41.97). In a small cohort such as ours, these observations should be interpreted cautiously; nevertheless, the presence of broader spectra in bundles may reflect less coordinated flagellar interactions, which would be consistent with elevated VCL but reduced VSL. The bundle B-E exemplified this, with a dominant high-frequency mode (3.69 Hz) and VSL = 6.92 µm/s. Visual analysis of the mode frequencies is depicted in Fig. 4, while mode amplitudes are depicted in Fig. 3. Kernel density estimation (KDE) of frequencies and amplitudes (see Fig. 5) suggests that bundles tend to have a wider range of frequencies and a narrower range of amplitudes. The dominant DMD frequency is expected to coincide with the primary peak obtained from a Fourier spectrum of local flagellar curvature, while additional harmonics may appear in the Fourier analysis. The dominant DMD frequency is expected to coincide with the primary peak obtained from a Fourier spectrum of local flagellar curvature, while additional harmonics may appear in the Fourier analysis.

#### Damping rates and movement efficiency

The damping rates (real parts of eigenvalues) further distinguished single cells from bundles. Single cells exhibited moderate damping ($\text {Re}(\lambda ) = -3.02$ to $-0.69$), allowing sustained oscillations for directional persistence. In contrast, bundles showed variable damping: B-B had strongly damped modes ($\text {Re}(\lambda ) = -0.52$ to $-1.44$), leading to transient oscillations, while B-A retained less damped modes ($\text {Re}(\lambda ) = -3.34$ to $-2.62$), enabling prolonged but non-progressive movement. This suggests that while bundles harness additive flagellar forces for speed (high VCL), inefficient damping and frequency synchronization limit their ability to convert motion into forward progression (low VSL).

#### Implications of bundle formation

The trade-off between speed and directionality in bundles may reflect an adaptive strategy for navigating viscous environments, where raw propulsion outweighs steering precision. However, the stark reduction in VSL highlights a functional limitation: without precise coordination, bundles sacrifice fertilization efficiency. The exceptional VSL of bundle B-A underscores the importance of structural cohesion in bundles, potentially mediated by biochemical or mechanical coupling. Future studies could explore whether external cues (e.g., shear stress, chemical gradients) enhance bundle coordination to improve VSL.

### Flagellar synchronization in sperm bundles

The question of whether sperm bundles achieve flagellar synchronization—a phenomenon critical for coherent collective motion—can be addressed through the integration of DMD-derived oscillatory dynamics and kinematic trends. Our analysis reveals that while bundles exhibit enhanced curvilinear velocity (VCL), their lack of progressive motility (VSL) and heterogeneous frequency spectra suggest limited synchronization.

#### Frequency discordance and asynchronous beating

In single cells, dominant low-frequency modes (e.g., 0.795 Hz in S-G and 1.346 Hz in S-A) with narrow bandwidths reflect stable, rhythmic flagellar beats conducive to directional swimming. In contrast, bundles displayed broader frequency distributions, often spanning multiple non-harmonic modes (e.g., bundle B-F: 1.83 Hz, 3.17 Hz, 4.57 Hz). Such spectral dispersion implies that individual flagella within bundles operate at distinct frequencies, generating interference that dissipates coordinated thrust. For instance, the bundle B-B exhibited competing modes at 1.815 Hz and 3.301 Hz, correlating with its chaotic motion (VSL = 0.653 µm/s despite VCL = 86.272 µm/s). This frequency discordance aligns with the “noisy swarm” model of collective motion, where unsynchronized actuators reduce net efficiency.

#### Amplitude suppression and destructive interference

While single cells sustained high-amplitude oscillations (e.g., 54.01 in S-G), bundles showed attenuated amplitudes (e.g., 16.04–41.97 in B-D), even at similar frequencies. This amplitude suppression suggests destructive mechanical interference between neighboring flagella. The bundle B-A—an outlier with relatively high VSL (14.52 µm/s)—featured a dominant mode at 2.819 Hz (amplitude = 41.97), hinting at partial synchronization. However, its secondary high-frequency mode (8.525 Hz, amplitude = 16.80) likely introduced instability, limiting VSL compared to single cells. These observations support the hypothesis that synchronization requires not only frequency alignment but also phase coherence, which is disrupted in most bundles by variable damping rates ($\text {Re}(\lambda ) = -0.52$ to $-3.34$).

#### Damping dynamics and transient coordination

The damping rates (real parts of eigenvalues) further contextualize synchronization challenges. Single cells exhibited lightly damped modes ($\text {Re}(\lambda ) \approx -0.69$ to $-3.02$), enabling persistent oscillations. Bundles, however, displayed stronger damping (e.g., $\text {Re}(\lambda ) = -3.34$ in B-A), causing rapid decay of oscillatory energy. This damping heterogeneity likely prevents sustained phase locking between flagella. For example, B-F showed heavily damped modes ($\text {Re}(\lambda ) = -0.52$ to $-1.44$), resulting in transient, self-terminating beats that preclude stable synchronization.

#### Structural vs. Functional coupling

The exception—B-A—suggests that mechanical coupling (e.g., via adhesive structures or fluid forces) may transiently align flagellar beats. Its dominant 2.819 Hz mode ($\text {Re}(\lambda ) = -2.62$) retained sufficient energy to sustain partial coordination, unlike other bundles. However, the absence of such behavior in most bundles implies that structural coupling alone is insufficient for synchronization; functional regulation (e.g., biochemical signaling or hydrodynamic feedback) may be required.

### Comparison of dynamic mode decomposition with other methods

Quantitative characterization of motile biological systems, such as sperm cell dynamics, requires analytical tools capable of extracting meaningful spatiotemporal information from high-dimensional and noisy experimental data. Among the most commonly employed approaches that can be compared to DMD are Fourier analysis, principal component analysis (PCA), and genetic algorithms (GA).

In the context of sperm cell motility, Fourier methods [60] are typically applied to the spatiotemporal deformation of the flagellum after transforming the data into a material reference frame attached to the sperm head [61, 62]. This approach enables direct estimation of beating frequency, wavelength, bending amplitude, and mean curvature.

The principal advantage of Fourier analysis lies in its computational efficiency and clear physical interpretation when the underlying motion is periodic and stationary. For single sperm cells exhibiting near-sinusoidal flagellar waves, low-order Fourier modes are often sufficient to capture the dominant dynamics. However, Fourier analysis relies on strong assumptions of stationarity, linear superposition, and global periodicity. These assumptions are frequently violated in biological systems, particularly under conditions involving transient behavior, asymmetric beating, or collective dynamics such as sperm bundling.

Moreover, by construction, Fourier analysis removes translational and rotational motion through the use of a sperm-centered reference frame. As a result, whole-cell swimming dynamics and directional motion are excluded from the analysis, limiting the method’s ability to capture the full kinematic behavior of motile cells.

PCA [63] is particularly effective for noise reduction and data compression and is often used as a preprocessing step prior to further analysis. Unlike Fourier analysis, PCA can capture asymmetric and non-periodic spatial features. However, PCA is inherently variance-based and does not incorporate temporal causality or dynamics. The extracted modes are not associated with characteristic frequencies, growth rates, or stability properties, and their temporal coefficients do not correspond to dynamical eigenvalues. PCA provides limited insight into the underlying physical or dynamical mechanisms governing motion. While it identifies *dominant shapes*, it does not explain how these shapes evolve in time or interact dynamically.

In the analysis of sperm motility, GA [64] are commonly employed to fit parametric biomechanical models of flagellar beating by optimizing physically meaningful parameters such as wavelength, amplitude, curvature, beating period, phase shift, and swimming direction [60].

A key strength of GA lies in its ability to recover interpretable biomechanical parameters while explicitly incorporating sperm translation, rotation, and hydrodynamic effects. GA do not require differentiability of the objective function and are robust against noise and nonlinearities, making them well suited for complex experimental data.

Nevertheless, GA are inherently model-dependent. The validity of the results is constrained by the correctness of the assumed physical model. They are computationally expensive, require careful tuning of hyperparameters, and are less suited for exploratory analysis. GA address parameter estimation rather than the discovery of emergent dynamical structures.

Unlike PCA, DMD explicitly incorporates temporal information, enabling identification of coherent dynamical structures and oscillatory behavior. Unlike Fourier analysis, DMD does not require stationarity or predefined basis functions and can capture transient and non-periodic dynamics. In contrast to GA, DMD does not assume an explicit physical or biomechanical model.

On the contrary, DMD is particularly effective for analyzing synchronization phenomena, mode coupling, and stability in complex motile systems. However, its modes are mathematically defined and do not directly correspond to physical parameters such as forces or energetic costs. Additionally, the linear approximation inherent in DMD may limit its accuracy for strongly nonlinear systems.

#### Comparative perspective

The four methods discussed address fundamentally different analytical objectives. Fourier analysis is best suited for clean, periodic single-cell motion; PCA excels at dimensionality reduction and dominant shape extraction; GA provide physically interpretable parameter estimation when a reliable model is available; and DMD offers a powerful framework for uncovering dominant spatiotemporal dynamics without strong a priori assumptions.

Rather than being mutually exclusive, these approaches are complementary. Hybrid workflows that combine statistical decomposition (PCA), dynamical mode extraction (DMD), and model-based optimization (GA) offer a promising strategy for achieving both descriptive and mechanistic understanding of complex biological motility.

## Conclusion

In this study, we employed DMD to analyze the motility patterns of single sperm cells and sperm bundles. By decomposing the time-resolved video data into dynamic modes, we identified the dominant periodic modes that characterize the flagellar beating patterns essential for propulsion.

This study demonstrates that sperm bundles achieve higher velocities at the cost of directional efficiency, governed by distinct DMD-derived frequency and damping dynamics. Single cells optimize progressive motility through low-frequency, rhythmic beats, while bundles leverage multi-flagellar forces for speed; however, with compromised steering. These findings advance our understanding of collective sperm behavior and its implications for reproductive success.

The data reveal that most sperm bundles lack consistent flagellar synchronization under the observed conditions. Their elevated VCL arises from the additive propulsion of individual cells, while poor VSL stems from frequency discordance, destructive interference, and transient damping. However, we emphasize that the present study is based on a small cohort (7 single cells and 6 bundles), which limits statistical power; consequently, conclusions about the prevalence of synchronization and the interpretation of outliers (e.g., B-A) should be treated as preliminary. We therefore frame the present work as a proof-of-concept study demonstrating that DMD can be applied to time-resolved sperm microscopy image sequences to extract interpretable frequency and damping characteristics.

In ongoing and future work, we plan to increase the sample size substantially (targeting $N>20$ per group) and to expand the conditions studied (e.g., viscosity, incubation time, and external cues such as shear flow or chemical gradients) to test the robustness and generalizability of the observed trends.

The main advancement of this work lies in demonstrating the applicability of DMD to biological microswimmers, specifically sperm cells. This approach provides a quantitative framework for dissecting the complex spatiotemporal dynamics of sperm motility without requiring prior knowledge of the underlying biological mechanisms. The novelty of our study is highlighted by the ability to capture and interpret the enhanced propulsion efficiency in sperm bundles due to flagellar synchronization, a phenomenon primarily driven by hydrodynamic interactions.

The application potential of the above-described findings extends to several domains. In reproductive biology, the quantitative assessment of sperm motility patterns can improve diagnostic techniques for male fertility by identifying anomalies in flagellar function and synchronization. Enhancing sperm selection processes based on motility characteristics may increase the success rates of *in vitro* fertilization and other fertility treatments. Furthermore, insights into the efficient propulsion mechanisms of sperm bundles can inform the design of sperm-inspired microrobots for biomedical applications such as targeted drug delivery and minimally invasive surgery.

## Supplementary Information

Below is the link to the electronic supplementary material.

Supplementary file 1 (pdf 1260 KB)

## Data Availability

No datasets were generated or analysed during the current study.
