# Supplementary material for: Elucidating motion patterns in sperm cell motion with dynamic mode decomposition
Source: J Biol Phys. 2026 Mar 27;52(1):15. doi: 10.1007/s10867-026-09710-3 (PMC13031697; doi:10.1007/s10867-026-09710-3)
Supplement: Supplementary file 1 — (pdf 1260 KB) [file 10867_2026_9710_MOESM1_ESM.pdf]

Supplementary Materials

Mode	Amplitude	Continuous Eigenvalue (Real: s^{-1}, Imag: rad s^{-1})	Frequency (Hz)
2	18.348	$-3.021 \pm 8.459j$	1.346
4	17.247	$-6.709 \pm 22.187j$	3.531
6	15.001	$-1.140 \pm 16.650j$	2.650
8	10.731	$-0.689 \pm 32.175j$	5.121

Table S1: Single cell 1.

Mode	Amplitude	Continuous Eigenvalue (Real: s^{-1}, Imag: rad s^{-1})	Frequency (Hz)
2/3	25.876	$-3.932 \pm 19.635j$	3.125
4/5	13.270	$-1.106 \pm 27.647j$	4.400
7/8	3.072	$-4.530 \pm 51.734j$	8.234
9/10	2.873	$-2.263 \pm 73.654j$	11.722

Table S2: Single cell 2.

Mode	Amplitude	Continuous Eigenvalue (Real: s^{-1}, Imag: rad s^{-1})	Frequency (Hz)
1/2	28.834	$-2.974 \pm 16.879j$	2.686
5/6	12.834	$-1.746 \pm 24.445j$	3.891
7/8	10.510	$-3.362 \pm 48.075j$	7.651
9/10	10.114	$-2.426 \pm 36.147j$	5.753

Table S3: Single cell 3.

PCA/Fourier/DMD comparison

As part of the Supplementary Information (SI), we have selected a video of a single sperm cell to compare the outputs of Fourier analysis, Principal Component Analysis (PCA) and Dynamic Mode Decomposition (DMD).

Principal Component Analysis (PCA)

PCA can capture asymmetric and non-periodic spatial features. In Figure S4 you can see how much information each mode contains. In Figure S5 you can see the spatial dynamics of top six modes. Also Fast Fourier Transform has been applied to each mode to elucidate the frequency spectrum.

Mode	Amplitude	Continuous Eigenvalue (Real: s^{-1}, Imag: rad s^{-1})	Frequency (Hz)
3/4	24.348	$-3.462 \pm 24.090j$	3.834
5/6	19.642	$-0.518 \pm 9.124j$	1.452
7/8	16.180	$-2.171 \pm 19.808j$	3.153
9/10	8.446	$-1.167 \pm 35.581j$	5.663

Table S4: Single cell 4.

Mode	Amplitude	Continuous Eigenvalue (Real: s^{-1}, Imag: rad s^{-1})	Frequency (Hz)
3/4	18.196	$-1.458 \pm 8.366j$	1.332
5/6	16.572	$-0.008 \pm 14.579j$	2.320
7/8	6.273	$-1.907 \pm 36.146j$	5.753
9/10	4.393	$-0.943 \pm 32.550j$	5.181

Table S5: Single cell 5.

Mode	Amplitude	Continuous Eigenvalue (Real: s^{-1}, Imag: rad s^{-1})	Frequency (Hz)
3/4	17.787	$-0.534 \pm 12.547j$	1.997
5/6	16.279	$-2.713 \pm 31.199j$	4.966
7/8	16.259	$-3.841 \pm 44.049j$	7.011
9/10	13.770	$-1.072 \pm 23.224j$	3.696

Table S6: Single cell 6.

Fourier analysis

As mentioned in our paper, performing the Fourier analysis on raw data comes with significant assumptions of stationarity, linear superposition, and global periodicity which in the case of this video are violated. The results of the analysis can be seen in Figure S6

Dynamic Mode Decomposition (DMD)

In Figure S7 and Table S14 you can see the full results of DMD analysis performed on a single sperm cell. Some modes visible in the table have been removed from the figure as they are associated with noise.

Mode	Amplitude	Continuous Eigenvalue (Real: s^{-1}, Imag: rad s^{-1})	Frequency (Hz)
3/4	54.008	$-1.583 \pm 4.994j$	0.795
5/6	21.567	$-1.031 \pm 14.721j$	2.343
7/8	12.825	$-0.268 \pm 20.917j$	3.329
9/10	11.651	$-0.765 \pm 33.113j$	5.270

Table S7: Single cell 7.

Mode	Amplitude	Continuous Eigenvalue (Real: s^{-1}, Imag: rad s^{-1})	Frequency (Hz)
5/6	41.965	$-2.618 \pm 17.713j$	2.819
7/8	17.151	$-3.289 \pm 8.577j$	1.365
9/10	16.803	$-3.338 \pm 53.565j$	8.525

Table S8: Bundle A

Mode	Amplitude	Continuous Eigenvalue (Real: s^{-1}, Imag: rad s^{-1})	Frequency (Hz)
3/4	37.304	$-0.572 \pm 11.403j$	1.815
7/8	26.194	$-1.106 \pm 20.738j$	3.301

Table S9: Bundle B

Mode	Amplitude	Continuous Eigenvalue (Real: s^{-1}, Imag: rad s^{-1})	Frequency (Hz)
3/4	34.360	$-0.671 \pm 6.812j$	1.084
6/7	18.600	$-0.358 \pm 12.963j$	2.063

Table S10: Bundle C

Mode	Amplitude	Continuous Eigenvalue (Real: s^{-1}, Imag: rad s^{-1})	Frequency (Hz)
6/7	24.964	$-0.982 \pm 11.081j$	1.764
8/9	19.288	$-4.808 \pm 18.537j$	2.950
10/11	16.036	$-1.127 \pm 28.641j$	4.558

Table S11: Bundle D

Mode	Amplitude	Continuous Eigenvalue (Real: s^{-1}, Imag: rad s^{-1})	Frequency (Hz)
1/2	49.823	$-4.305 \pm 18.263j$	2.907
6/7	28.923	$-1.827 \pm 23.203j$	3.693

Table S12: Bundle E

Modes	Amplitude	Continuous Eigenvalue (Real: s^{-1}, Imag: rad s^{-1})	Frequency (Hz)
4/5	19.759	$-0.521 \pm 11.517j$	1.833
6/7	19.543	$-1.440 \pm 19.933j$	3.172
8/9	13.015	$-0.913 \pm 28.719j$	4.571

Table S13: Bundle F

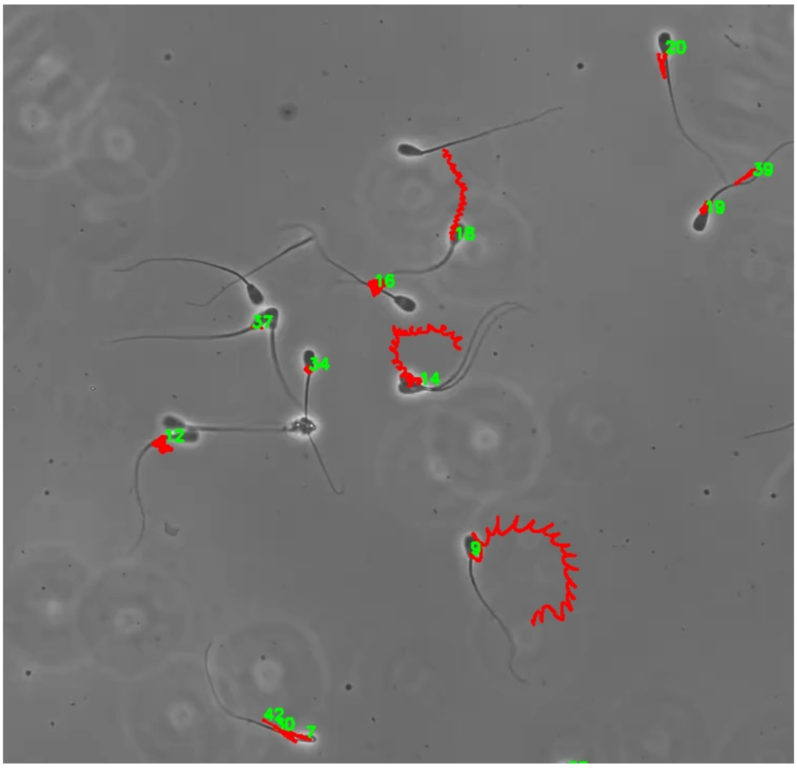

Fig. S1: Representative example of tracked cell trajectories. The bundle is labeled as ID 14.

mode	Amplitude	Discrete Eigenvalue	Continuous Eigenvalue	Frequency (Hz)	Magnitude
1	95.142	0.9849 - 0.0051j	-1.5189 - 0.5199j	0.083	0.9849
2	95.142	0.9849 + 0.0051j	-1.5189 + 0.5199j	0.083	0.9849
3	21.751	0.9722 - 0.0538j	-2.6670 - 5.5310j	0.880	0.9737
4	21.751	0.9722 + 0.0538j	-2.6670 + 5.5310j	0.880	0.9737
5	18.707	0.9830 - 0.1252j	-0.9074 - 12.6673j	2.016	0.9910
6	18.707	0.9830 + 0.1252j	-0.9074 + 12.6673j	2.016	0.9910
7	16.093	0.0035 + 0.0000j	-566.6539 + 0.0000j	0.000	0.0035
8	12.049	0.9436 + 0.2881j	-1.3488 + 29.6321j	4.716	0.9866
9	12.049	0.9436 - 0.2881j	-1.3488 - 29.6321j	4.716	0.9866

Table S14: Sperm cell complete information about the DMD modes.

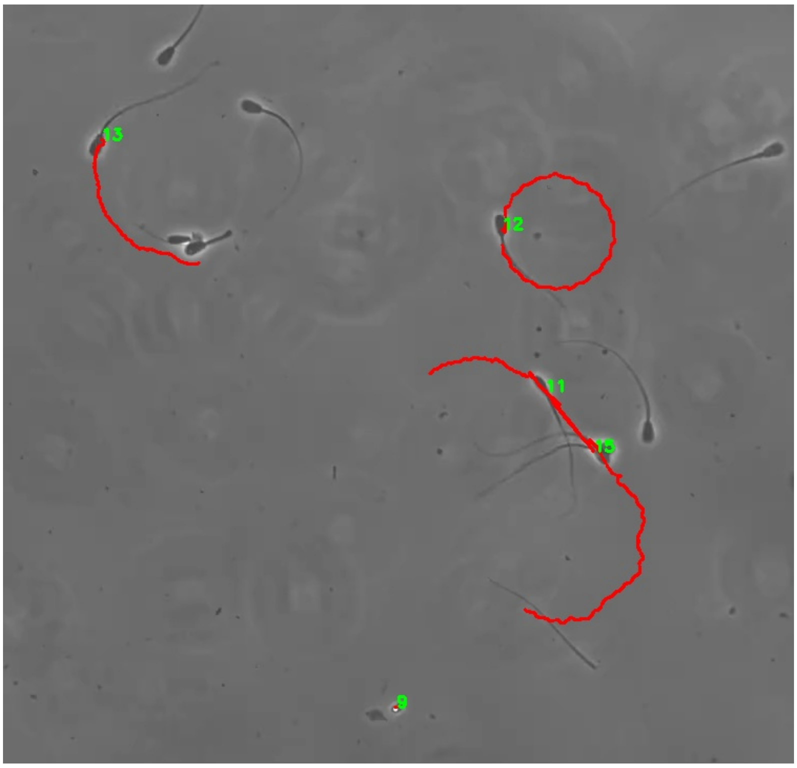

Fig. S2: Representative example of tracked cell trajectories. The bundle is labeled as ID 15.

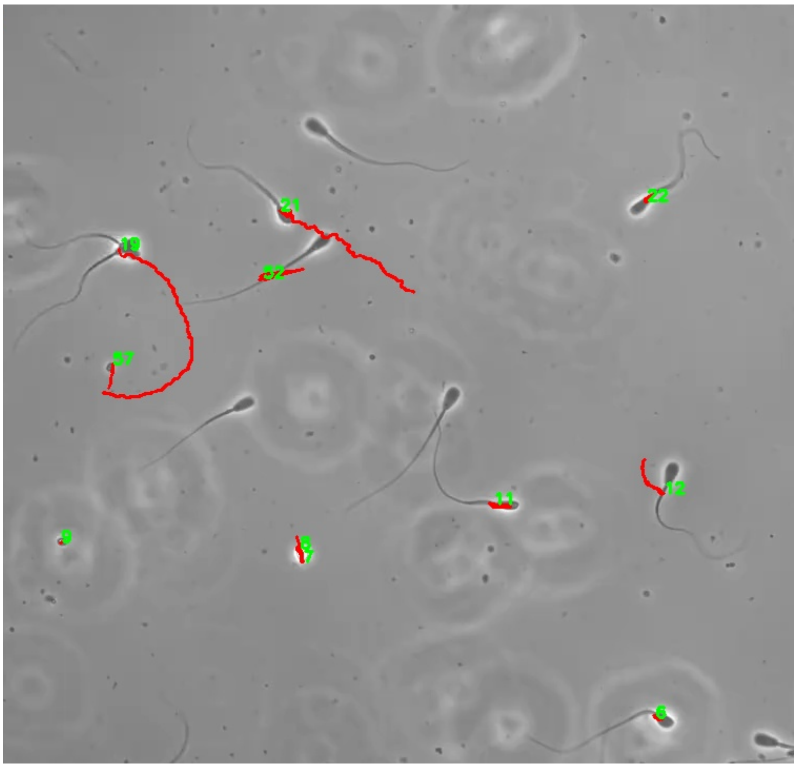

Fig. S3: Representative example of tracked cell trajectories. The bundle is labeled as ID 19.

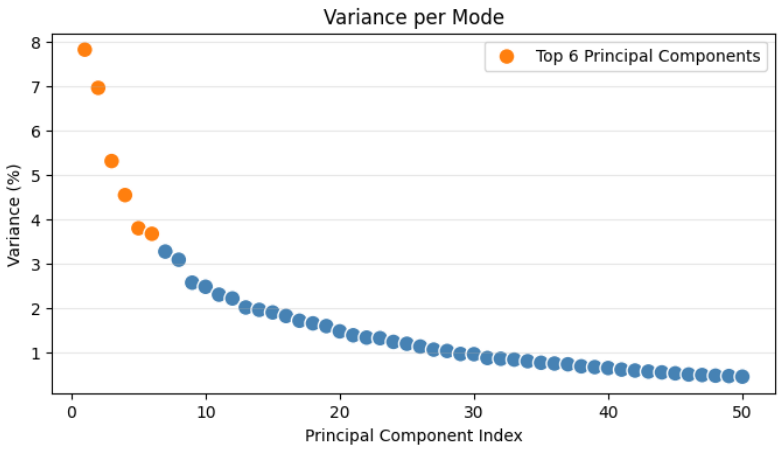

Fig. S4: PCA modes variance

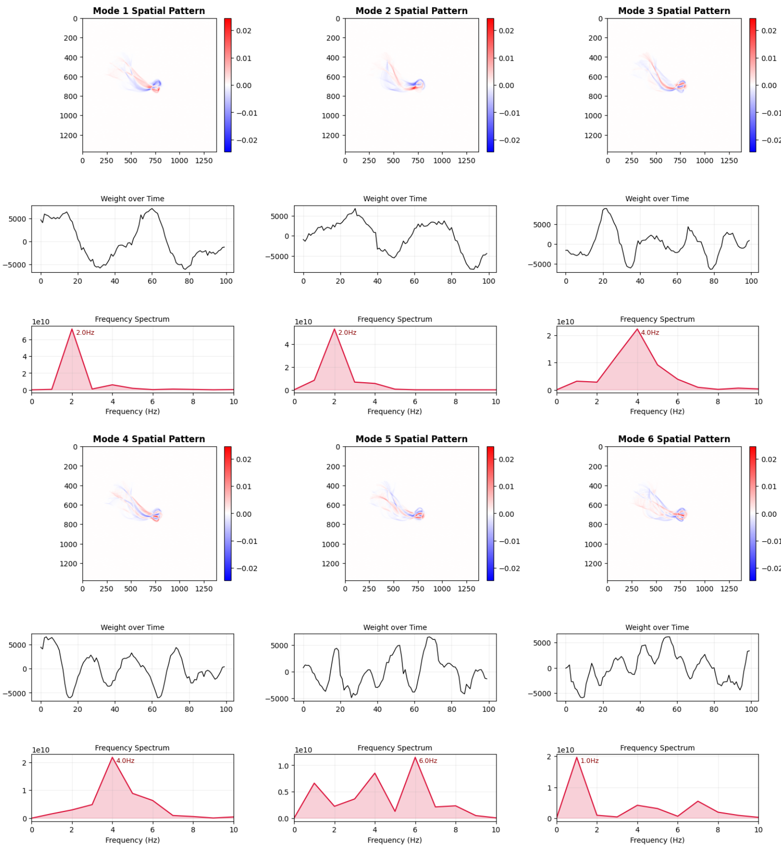

Fig. S5: PCA spatial modes

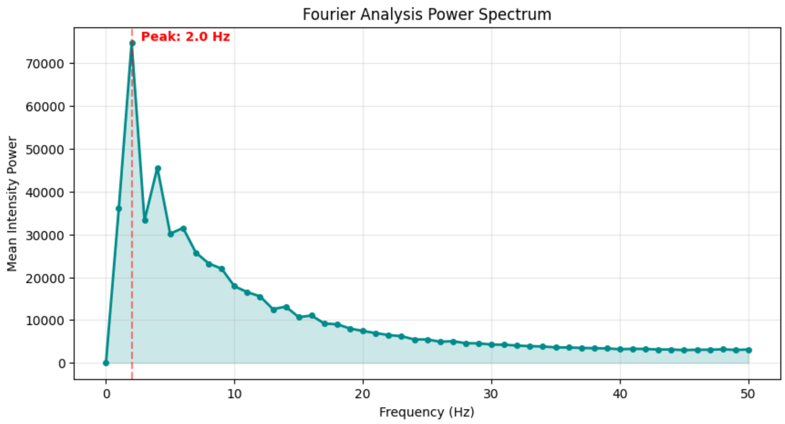

Fig. S6: Fourier analysis performed on raw data.

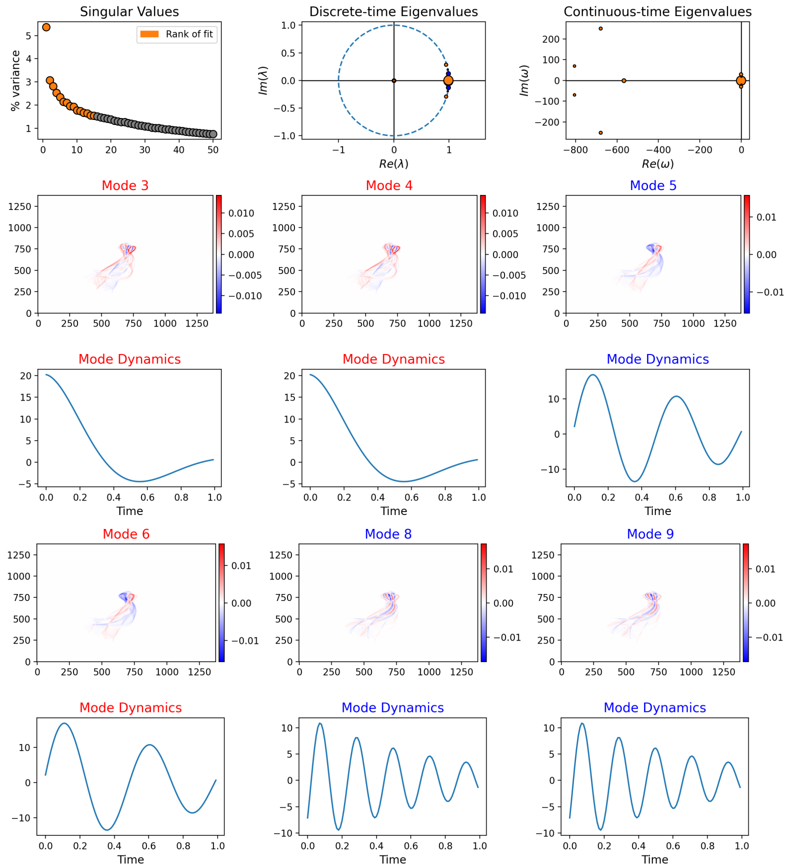

Fig. S7: Results of DMD Analysis
